# Functional Hydrogels Promote Vegetable Growth in Cadmium-Contaminated Soil

**DOI:** 10.3390/gels10050348

**Published:** 2024-05-20

**Authors:** Jin Huang, Takehiko Gotoh, Satoshi Nakai, Akihiro Ueda

**Affiliations:** 1 Graduate School of Advanced Science and Engineering, Hiroshima University, 1-4-1 Kagamiyama, Higashi-Hiroshima 739-8527, Hiroshima, Japan; hj520618618@outlook.com (J.H.); sn4247621@hiroshima-u.ac.jp (S.N.); 2 Graduate School of Integrated Sciences for Life, Hiroshima University, 1-4-1 Kagamiyama, Higashi-Hiroshima 739-8527, Hiroshima, Japan

**Keywords:** hydrogel, heavy metal, cadmium immobilize, soil

## Abstract

Over the years, the concentration of cadmium in soil has increased due to industrialization. Cadmium in the soil enters the human body through plant accumulation, seriously endangering human health. In the current study, two types of hydrogels were successfully synthesized using a free radical polymerization method: an ion-type hydrogel referred to as DMAPAA (*N*-(3-(Dimethyl amino) propyl) acrylamide)/DMAPAAQ (*N*,*N*-Dimethyl amino propyl acrylamide, methyl chloride quaternary) and a non-ion-type hydrogel known as DMAA (*N*,*N*-Dimethylacrylamide). In the experiment carried out in this study, the ion-type hydrogel DMAPAA/DMAPAAQ was introduced to cadmium-contaminated soil for vegetable cultivation. The study found that at cadmium levels of 0 and 2 mg/kg in soil, when exposed to a pH 2 solution, cadmium wasn’t detected in the filtrate using ICP. As the amount of cadmium increased to 500 mg/kg, hydrogel addition gradually reduced the filtrate cadmium concentration. Notably, the use of the 4% hydrogel resulted in 0 mg/L of cadmium. For the 0% hydrogel, vegetable cadmium absorption was determined to be 0.07 mg/g, contrasting with 0.03 mg/g for the 4% hydrogel. The DMAPAA/DMAPAAQ hydrogel significantly boosts vegetable growth by efficiently absorbing nitrate ions through ion exchange, releasing them for plant uptake. In contrast, the DMAA hydrogel, used as a control, does not enhance plant growth despite its water absorption properties. In summary, the composite hydrogel shows great potential for enhancing vegetable yield and immobilizing heavy metals in soil.

## 1. Introduction

The escalating process of global industrialization has given rise to a concerning surge in the presence of heavy metals in soil [1,2]. Among these metals, cadmium, a member of the second group of the periodic table, stands out for its pronounced toxicity and classification as one of the most hazardous elements. While the concentration of cadmium in natural soil has a limited impact on human health, anthropogenic activities such as atmospheric pollution, sewage irrigation, and excessive use of chemical fertilizers have led to the accumulation of cadmium in soil, heightening the risk it poses to human health [3,4]. Recent scientific reports reveal that heavy metal pollution has affected approximately 500,000 sites in Europe and over 60,000 hectares of land in the United States [5,6,7]. Given that heavy metals enter the food chain through soil and plant uptake, they present a severe threat to human health. Consequently, maintaining unpolluted soil is paramount for safeguarding the overall ecological environment and ensuring human well-being [8].

Addressing cadmium contamination in soil has underscored the growing importance of soil remediation techniques. Various methods, including physical, chemical, and biological approaches, have been employed, utilizing a diverse range of materials such as household discards, nanomaterials, and biomass materials. Among these, living discards such as eggshells, shells, oysters, and oyster shells have garnered attention for their calcium content, offering a potential substitute for cadmium in plants [9,10,11]. Research has demonstrated that the addition of shell powder to cadmium-contaminated soil can elevate soil pH and diminish cadmium accumulation in crops, as exemplified by the synergistic application of biochar and oyster shell [12,13]. For instance, introducing coconut shell biochar into soil alongside earthworms resulted in the removal of 94.38% of total cadmium from soil samples [14]. These findings highlight the promise of these materials as effective tools for remediating cadmium-contaminated soil.

The detrimental impact of cadmium contamination in soil has spurred the exploration of novel materials for its remediation, with nanoparticles gaining significant attention due to their substantial specific surface area and numerous surface-active sites [15,16]. For example, Watanabe et al. applied zero-valent iron to Cd-contaminated rice fields, observing a 10–20% decrease in Cd concentrations in seeds and leaves [17]. Similarly, Natasha Manzoor et al. utilized FeO nanoparticles to treat Cd-contaminated soil, resulting in a notable 72.5% reduction in Cd uptake by wheat plants [18]. CuO nanoparticles have also exhibited potential in mitigating Cd toxicity, with low concentrations (5 mg/L) leading to reduced Cd uptake in rice and barley plants [19]. Additionally, the use of zinc sulfate has demonstrated a 19–28% reduction in Cd concentrations in spinach leaves and a substantial 42% reduction in roots [20]. These findings underscore the efficacy of nanoparticles and other materials in remediating Cd-contaminated soil.

Biomass, derived from organic matter through photosynthesis, encompasses a diverse array of materials, including plants, animals, their excreta, garbage, and organic wastewater. The presence of hydroxyl and carboxyl groups in biomass materials renders them excellent adsorbents for heavy metals. For example, sludge, rich in organic materials and soluble ions, can effectively enhance soil pH. Bovine manure biochar, characterized by its porous structure and negatively charged surface, has demonstrated efficacy in removing heavy metals from soil through electrostatic adsorption [21,22]. Field experiments conducted by Atsushi Sato et al. showed that the addition of animal manure resulted in a 34–38% reduction in cadmium concentration in spinach compared to the use of chemical fertilizers [21]. Research by Evanise Silva Penido et al. revealed that the addition of wood biochar and biochar derived from sewage sludge to soil increased soil pH, effectively mitigating the impact of cadmium contamination [23]. These studies underscore the potential of various biomass materials in the remediation of cadmium-contaminated soil.

While various materials show promise in mitigating cadmium contamination in soil, certain limitations hinder their widespread application. Some materials are not produced on a large scale, and their adsorption of cadmium may alter its form in the soil without complete removal. Moreover, these materials often serve a singular function, primarily fixing cadmium without actively promoting plant growth. Additionally, under conditions such as acid rain or elevated carbon dioxide concentrations, the form of cadmium may change, potentially leading to its release into the environment [24,25]. The emergence of superabsorbent gel as a novel polymer material for soil remediation presents a promising solution. Its hydrophilic three-dimensional mesh structure enables it to absorb and retain water solutions up to hundreds of times its weight. Beyond its capacity to adsorb heavy metals in the soil, this gel improves soil moisture, providing plants with ample water to expedite their growth cycles [26,27]. Studies have highlighted the potential effectiveness of this material in mitigating cadmium contamination in soil. For instance, Liu et al. developed a ferrous sulfide (FeS) nanoparticle lignin hydrogel composite with a Cd adsorption capacity of 833.3 g/kg. Wei et al. applied FeS@LH to Cd-contaminated rice fields, resulting in the removal of 36.6% of Cd in soil and 34.5% of cadmium in water spinach after 30 days of treatment [28,29]. Zheng et al. created a versatile hydrogel that releases pesticides for controlling vegetable growth and can absorb heavy metals. The hydrogel showed high adsorption capacity for Cu (II) and Pb (II) [30]. He et al. incorporated coal ash-modified TOB into a starch/acrylic acid hydrogel, displaying notable adsorption capacity for cadmium [31]. In a another study, a composite hydrogel (AM/CMC/B) using peanut shells showed a maximum adsorption capacity for Cd^2+^ and demonstrated potential in remediating cadmium-contaminated soil, reducing soil inhibitory effects on tobacco seedlings [32].

In this study, two types of hydrogels, namely DMAPAA/DMAPAAQ and DMAA, were successfully synthesized using a free radical polymerization method. The main objective of this study was to explore the effects of incorporating these hydrogels into soil and comprehensively evaluate the growth of vegetables under cadmium stress or without cadmium. Specifically, the focus was on investigating the impact of the addition of the DMAPAA/DMAPAAQ ionic hydrogel on vegetable growth under cadmium stress, as well as the effects of the DMAA non-ionic hydrogel and the DMAPAA/DMAPAAQ ionic hydrogel on vegetable growth without cadmium. A comprehensive assessment of the influence of these two hydrogels on plant growth was carried out by measuring the dry weight of the vegetables. Additionally, the amount of cadmium absorbed by the vegetables was evaluated when the DMAPAA/DMAPAAQ ionic hydrogel was added to the soil under cadmium stress. Finally, an in-depth study on the content of elements in plants was carried out under the combined influence of hydrogels and cadmium. This comprehensive research aims to provide a deeper understanding of the application of hydrogels in vegetable cultivation and to provide scientific evidence for regulating plant growth under cadmium stress in soil.

## 2. Results and Discussion

### 2.1. Detection of Elements in Soil

**Figure 1:**
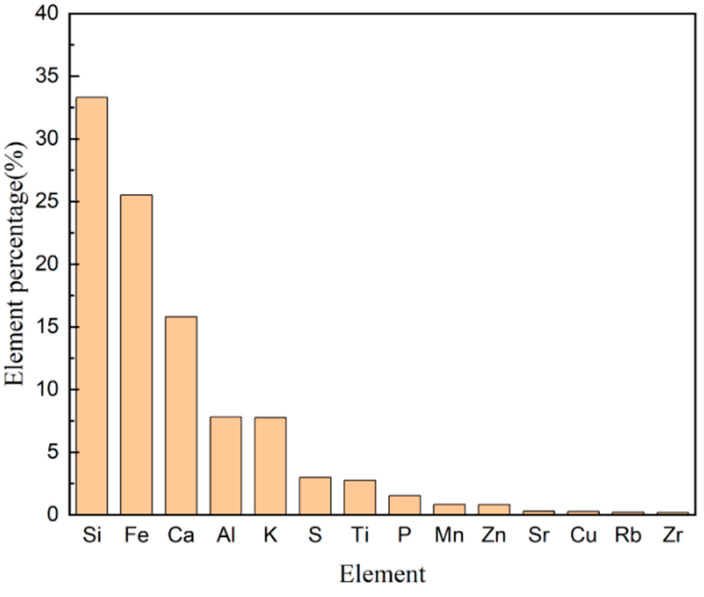
Elements in soil.

For elemental composition analysis of the soil, a detection method using an EDX-7000 energy dispersive XRF spectrometer was employed, and the detection results are illustrated in Figure 1. Silicon and iron emerged as the predominant elements in the soil samples, collectively constituting over 50% of the total composition. It is noteworthy that the presence of manganese and iron can exert an influence on the availability of cadmium in the soil. This influence is facilitated by the oxidation process of these elements, resulting in the formation of manganese oxide (MnO_2_/Mn_2_O_3_) and iron oxide (Fe (OH)_3_/FeOOH) [33]. During this process, heavy metal ions of cadmium co-precipitate with manganese oxide and iron oxide, impeding their release from the soil and consequently diminishing the toxicity of cadmium to plants. Moreover, silicon reacts with cadmium, leading to the formation of cadmium silicate precipitates [34]. This reaction effectively diminishes the content of highly active exchangeable cadmium in the soil, thereby mitigating its biological toxicity. This series of processes contributes to the optimization of the soil environment, ultimately enhancing the health of the plant growth environment. In addition, many elements in the soil are incompatible, which is not conducive to plant absorption. In the future, the combination of hydrogels and microorganisms could be employed to dissolve elements in the soil, making them available for plant absorption [35].

### 2.2. Soil pH

**Figure 2:**
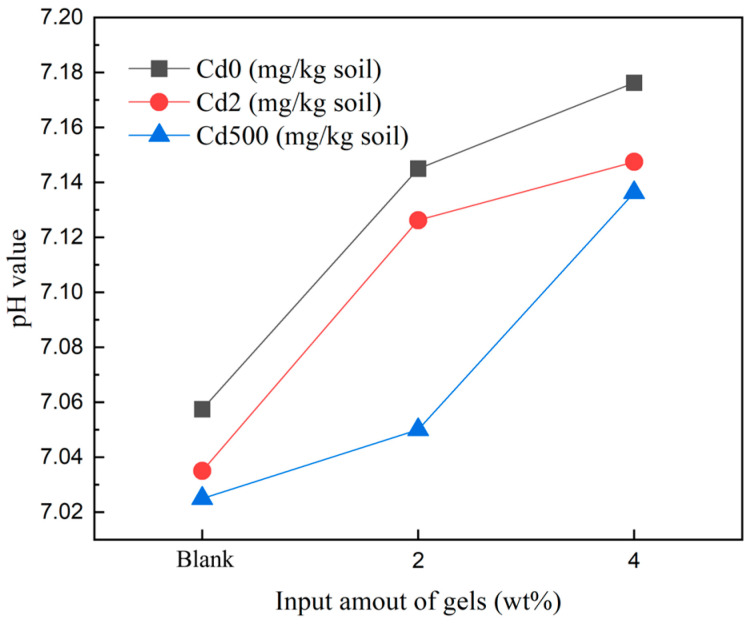
pH value of the soil.

The pH value of the soil, depicted in Figure 2 and measured using a pH meter, exhibited a slight increase with the addition of the hydrogel, indicating that the introduction of the polymer contributes to an improvement in soil pH. The hydrogel, being a three-dimensional network polymer, possesses the ability to absorb water exceeding its own weight, thereby aiding in the maintenance of soil moisture. As the cadmium concentration in the soil increased, a slight decrease in pH was observed; this shows that the hydroxyl groups formed through the protonation of DMAPAA are consumed more by cadmium, resulting in a decrease in OH^–^ ions in the soil and a slight decrease in soil pH. However, overall, the variation in soil pH remained minimal, consistently maintaining a balanced state at pH 7.

### 2.3. Cadmium Precipitation Experiments in Soil

**Figure 3:**
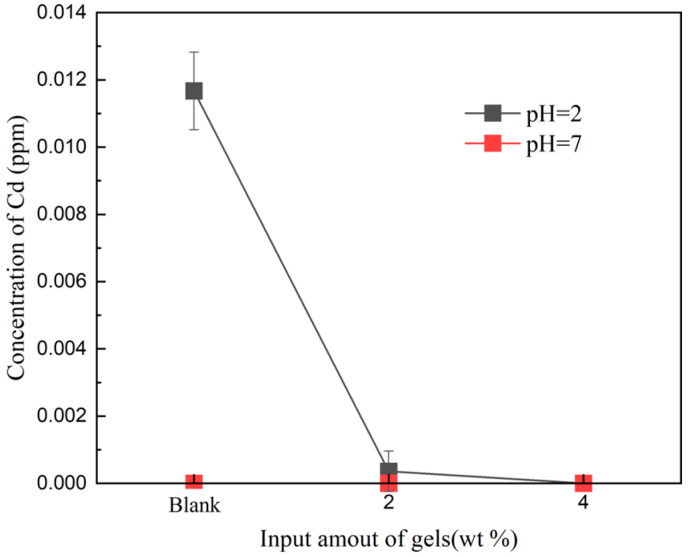
Cadmium precipitation values of soil in different pH solutions.

In an experiment examining cadmium desorption in soil with varying cadmium concentrations, the pH of the solution was adjusted across different conditions, as illustrated in Figure 3. In a solution with a pH of 2 and the cadmium concentration in the soil at 0 or 2 mg/kg, the cadmium concentration in the filtrate remained at 0 mg/L. However, when the cadmium concentration in the soil was elevated to 500 mg/kg, the cadmium concentration in the filtrate gradually decreased with an increase in the amount of gel added. In the control group without gel addition, the Cd concentration in the solution was 0.015 mg/L, whereas after adding 4% gel, the Cd concentration in the filtrate reached 0 mg/L. In a solution with a pH of 7, the desorption values of Cd in the soil were consistently 0. This suggests that at low cadmium concentrations, Si, Fe, and Mn in the soil can form insoluble compounds with cadmium. However, at high cadmium concentrations, the negative charge on the soil surface and certain metal colloids prevents the sufficient absorption of cadmium. The addition of gel proves effective in absorbing excess cadmium. DMAPAA water gel protonates its surface in water, generating hydroxyl groups that react with cadmium to form insoluble cadmium hydroxide precipitates, ultimately encapsulated by the gel.

### 2.4. Growth State of Vegetables under Cadmium Stress

**Figure 4:**
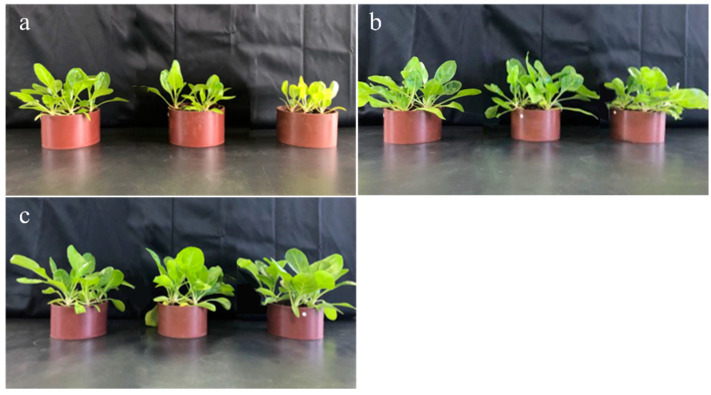
Images of vegetable growth: for images (**a**–**c**), the addition of hydrogel is at the 0%, 2%, and 4% level. From left to right, the amount of cadmium added is 0 mg/kg, 2 mg/kg, and 500 mg/kg.

The growth status of vegetables under cadmium stress was recorded using a camera after the addition of the DMAPAA/DMAPAAQ hydrogel to the soil (Figure 4). The results revealed a positive correlation between the growth condition of vegetables and the quantity of hydrogel added, suggesting a beneficial impact on vegetable growth under cadmium stress. The hydrogel serves two pivotal roles: firstly, its three-dimensional network structure absorbs a substantial amount of water, enhancing root respiration and promoting photosynthesis, thereby accelerating vegetable growth [36]. Secondly, the DMAPAA/DMAPAAQ composite hydrogel can adsorb cadmium, stabilizing more cadmium in the soil and significantly reducing the biotoxicity of cadmium to vegetables, ultimately improving their growth state.

### 2.5. Vegetable Growth Status under Two Types of Hydrogels

**Figure 5:**
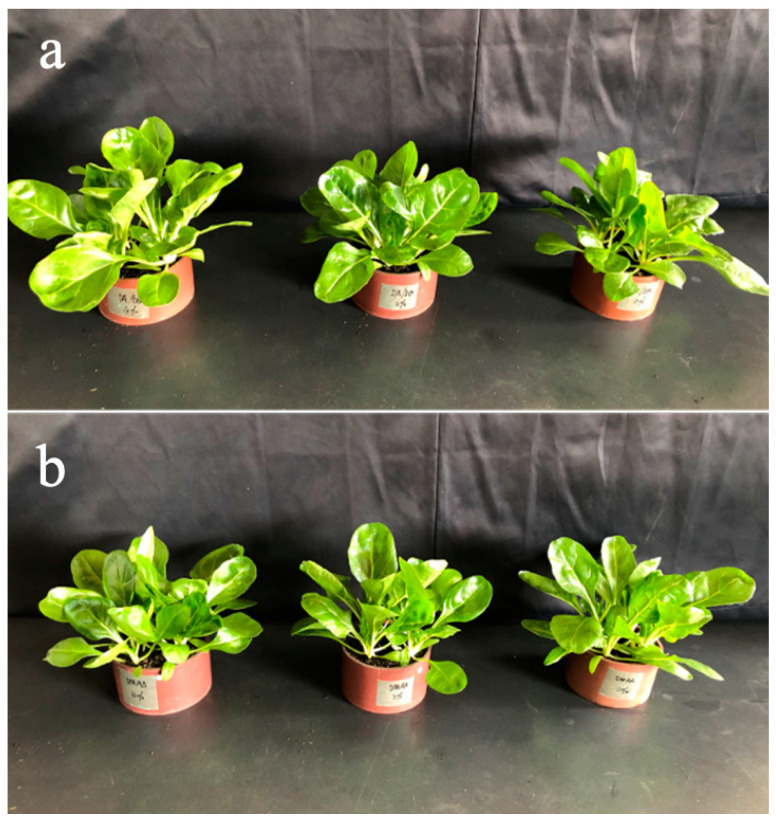
Physical image of vegetable growth: (**a**) from left to right, the DMAPAA/DMAPAAQ hydrogel addition amount is 4%, 2%, and 0%; (**b**) from left to right, the DMAA hydrogel addition amount is 4%, 2%, and 0%.

Experiments were conducted with two distinct control groups employing different types of hydrogels, namely DMAA and DMAPAA/DMAPAAQ hydrogels, with a focus on understanding their impact on plant growth under cadmium-free conditions (Figure 5). DMAA represents a non-ionic polymer, while DMAPAA/DMAPAAQ falls into the category of ionic polymers. Despite some structural similarities, with both containing amide groups and exhibiting the ability to absorb a substantial amount of water for soil moisture retention, these hydrogels differ in their functionalities. The experimental results clearly demonstrate that an increasing amount of DMAPAA/DMAPAAQ hydrogel leads to more vigorous vegetable growth, indicating a positive impact on plant development. Specifically, DMAPAA undergoes protonation in water, resulting in a positively charged surface capable of electrostatically adsorbing certain cations in the soil, such as K and Ca—soluble ions known to promote plant growth. Conversely, the DMAPAAQ hydrogel engages in the exchange of NO_3_^−^ and PO_4_^3−^ ions in the soil through ion exchange, subsequently releasing them gradually for plant absorption, further promoting plant growth [37]. In contrast, the DMAA hydrogel, utilized as a control, despite its water absorption properties, did not exhibit any notable promotion of plant growth based on the experimental results (see Figure S1). These findings offer valuable insights for selecting suitable hydrogels to enhance plant growth under cadmium-free conditions.

### 2.6. Cadmium Element Content in the Vegetables

**Figure 6:**
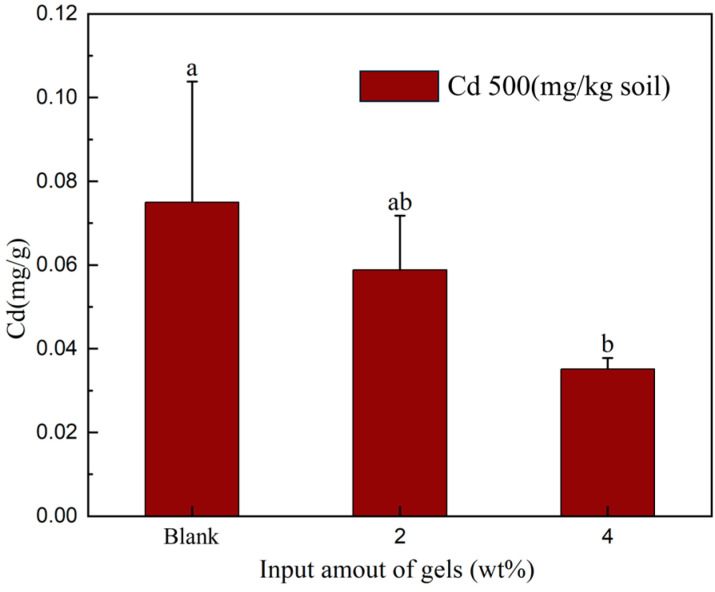
The amount of Cd absorbed by the studied vegetables.

Based on the ICP determination results depicted in Figure 6, an examination of the cadmium content in the vegetables was conducted. Notably, with the increasing amount of gel added, there was a gradual decrease in the absorption of cadmium by the vegetables. In the absence of gel addition, the cadmium absorption by the vegetables was measured at 0.07 mg/g, which is three times higher than the amount absorbed when 4% gel was added. However, with the addition of 4% gel, the accumulation of cadmium in the vegetables diminishes to only 0.03 mg/g. This observation underscores the hypothesis that the incorporation of the DMAPAA/DMAPAAQ hydrogel effectively reduces the absorption of cadmium by vegetables. Hence, the DMAPAA/DMAPAAQ hydrogel not only demonstrates efficacy in promoting plant growth but also exhibits significant potential in mitigating the absorption of harmful metal elements in soil. These findings provide valuable empirical support for the utilization of hydrogels in agricultural production, contributing to the enhancement of soil environmental quality.

### 2.7. Vegetable Uptake of Different Elements

**Figure 7:**
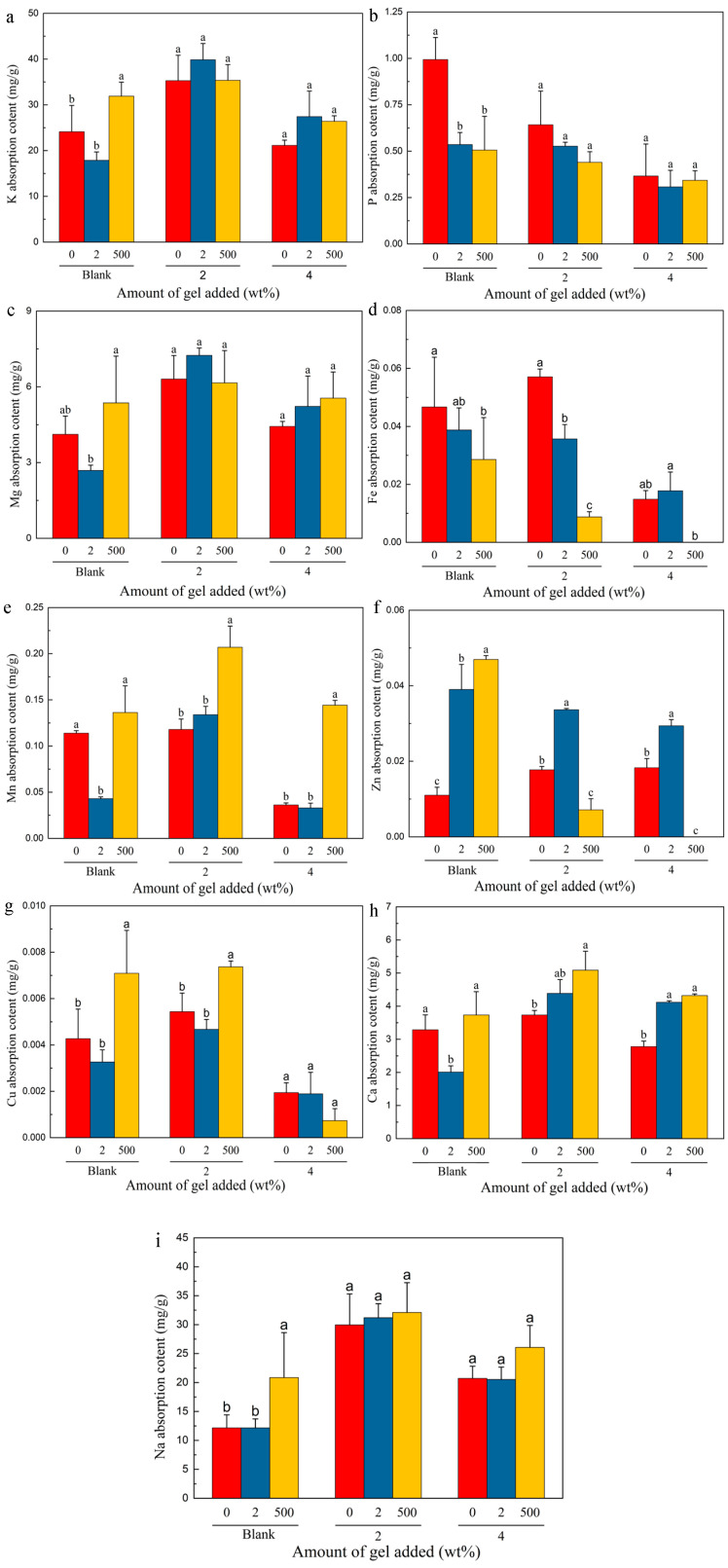
Contents of various trace elements in vegetable leaves: (**a**) potassium; (**b**) phosphorus; (**c**) magnesium; (**d**) iron; (**e**) manganese; (**f**) zinc; (**g**) copper; (**h**) calcium; (**i**) sodium.

ICP analysis was utilized to assess the concentrations of essential elements, including Na, Mg, K, Ca, P, Fe, Mn, Cu, and Zn, in the vegetables (Figure 7). These elements are pivotal for the normal growth and development of vegetables, serving as integral components of enzymes, vitamins, and hormones. Additionally, these elements serve as critical parameters for evaluating soil fertility. A substantial portion of the nutrient elements in plants originates from the soil, with soil organic matter being primarily derived from deceased organic materials of animals and plants. Over time, microorganisms break down these large organic molecules into smaller molecules or even inorganic substances, which are subsequently absorbed by plants. Consequently, the measurement of essential element concentrations in vegetables allows us to assess not only whether hydrogels facilitate nutrient transport to promote plant growth but also indirectly evaluate soil fertility. The results shown in Figure 7 indicate relatively high concentrations of K, P, Mg, and Na in the vegetables, suggesting corresponding elevated levels in the soil. Additionally, the addition of hydrogels aids in the absorption of these elements by plants, particularly benefiting the normal growth of plants, as elements such as K, P, and Mg are essential for most plant species. However, sodium is harmful to some plants, whereas in low concentrations, it is beneficial to C4 plants. By controlling the sodium concentration in the hydrogel, rational management of sodium can be achieved, making it an effective fertilizer within a certain concentration range. Therefore, considering different methods to optimize plant growth, sodium-containing hydrogels can be considered for application in agriculture to replace or supplement potassium and promote healthy plant growth [38]. Interestingly, despite soil having high iron content, the iron concentration in plants is not significant. This observation suggests that iron primarily exists in the soil in a non-ionic form rather than in an ionic form. Notably, the addition of hydrogels does not enhance the absorption of iron from the soil by plants, indicating that hydrogels may exert selective effects on the absorption of different forms of elements in the soil. These detailed observations provide valuable insights for a deeper understanding of the mechanisms through which hydrogels influence nutrient absorption in the soil and plant growth.

### 2.8. Hydrogel Repair Mechanism for Cadmium-Contaminated Soil

**Figure 8:**
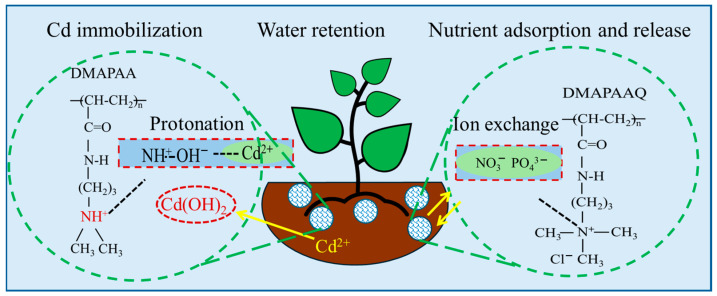
The removal mechanism of heavy metal cadmium by the DMAPAA/DMAPAAQ composite hydrogel. Yellow arrows indicate hydrogel ion exchange.

In the soil, manganese and iron undergo oxidation, giving rise to manganese oxide (MnO_2_/Mn_2_O_3_) and iron oxide (Fe (OH)_3_/FeOOH). This process facilitates the co-precipitation of specific heavy metal ions, including cadmium. Notably, iron, silicon, and cadmium in the soil possess chelating properties, allowing them to form Fe-Cd and Si-Cd structures, effectively immobilizing cadmium. DMAPAA/DMAPAAQ hydrogels, as ion-type polymers, play a pivotal role in these processes. Conversely, the DMAA hydrogel, functioning as a non-ionic polymer, operates differently. DMAPAA undergoes protonation in water, generating hydroxyl groups on its surface that enable the adsorption of metal cations. Table 1 shows a comparison of the adsorption of heavy metals between the adsorption materials DMAPAA/DMAPAAQ and other materials. The adsorption capacity of the DMAPAA/DMAPAAQ hydrogel adsorbent is significantly higher than that of other adsorbent materials. Subsequently, it forms hydroxide precipitates with heavy metals, encapsulating them within the hydrogel and providing an effective mechanism for cadmium immobilization. Additionally, DMAPAA can adsorb potassium and calcium in their ionic forms, forming soluble compounds that facilitate plant uptake. On the other hand, DMAPAAQ, as a cationic polymer, engages in the exchange of anions in the soil, such as NO_3_^−^ and PO_4_^3−^, through ion exchange (see Figure 8). Given that these anions are typically not adsorbed by the soil and are prone to leaching with rainfall, DMAPAAQ adsorbs them and gradually releases them for plant uptake, promoting plant growth. Simultaneously, the release of chloride ions by DMAPAAQ has a promoting effect on certain plants, such as corn. The mechanisms of action elucidated for these hydrogels highlight their versatility in regulating heavy metal ions and providing essential nutrient elements in the soil. This versatility positions them as a potential environmentally friendly solution for enhancing soil quality and promoting plant growth. In the future, the synergistic effect of hydrogels and microorganisms can be used to remediate soil contaminated by heavy metals. Microorganisms are a gentle adsorption method, which is different from chemical remediation which may bring secondary pollution to the soil. At the same time, hydrogels can absorb a large amount of water, which provides a good growth environment for microorganisms [39].

## 3. Conclusions

This study focused on the effects of two different types of hydrogels—DMAA nonionic hydrogel and DMAPAA/DMAPAAQ ionic hydrogel on vegetable growth in cadmium contaminated soil. The experimental results showed that after adding DMAPAA/DMAPAAQ hydrogel, the ability of soil to fix cadmium gradually increased, especially in high iron soil. When the concentration of hydrogel reaches 4% in the hydrochloric acid solution with pH 2 in the soil, the leaching value of cadmium in the soil can be reduced to zero, which proves that the hydrogel has an efficient fixation effect on cadmium. At the same time, when the concentration of cadmium is under the condition of low cadmium concentration when no hydrogel is added, the absorption of cadmium by vegetables cannot be detected. However, when the concentration of cadmium reached 500 mg/kg, when no hydrogel was added, the absorption of cadmium by vegetables reached 0.075 mg/kg; When 4% hydrogel was added, the absorption of cadmium by plants decreased to the minimum value of 0.03 mg/g. In contrast, ionic hydrogels show significant advantages in vegetable cultivation, which can effectively absorb nutrients such as nitrate ions and phosphate ions that are not easily fixed in the soil, and slowly release these nutrients through ion exchange to promote the growth of vegetables. On the contrary, the use of non-ionic hydrogel DMAA led to a decrease in the dry weight of vegetables. In general, the research results emphasize the potential application of ionic hydrogels in soil, which can not only promote plant growth, but also effectively immobilize heavy metal cadmium. Future research may explore safe and non-toxic natural ionic polymers that can absorb heavy metals while providing essential nutrients for plants.

## 4. Materials and Methods

### 4.1. Materials

The monomer DMAPAA/DMAPAAQ was obtained from KJ Chemicals Corporation (Tokyo, Japan), while *N*,*N*-dimethyl ethylenediamine (TEMED), HNO_3_, and H_2_O_2_ were procured from Nacalai Tesque Co., Ltd. (Kyoto, Japan). DMAA was purchased from Tokyo chemical industry Co., LTD. (Tokyo, Japan). *N*,*N*′-methylenebisacrylamide (MBAA), ammonium persulfate (APS), CdN_2_O_8_·4H_2_O, and CdCl_2_·2.5H_2_O were obtained from Sigma-Aldrich, Inc. (St. Louis, MO, USA). Additionally, 1 mol/L HCl solutions were purchased from Sigma-Aldrich Japan (Tokyo, Japan). The soil used in this study was acquired from NAFCO (Fukuoka, Japan). All reagents utilized were of analytical grade and used as received. Distilled water for the experiments was produced in the laboratory.

### 4.2. Synthesis of the DMAPAA/DMAPAAQ Hydrogel

To synthesize the hydrogel, 1.953 g of DMAPAA and 2.584 g of DMAPAAQ monomers were precisely weighed, along with 0.193 g of MBAA crosslinker and 0.058 g of TEMED accelerator. These components were combined in a 20 mL volumetric flask (refer to Table 2). Distilled water was then added to the flask, and the resulting mixture was stirred using a magnetic stirrer. In a separate 5 mL volumetric flask, a solution containing 0.114 g of APS initiator was prepared using distilled water. Both the monomer solution and the initiator solution underwent a 45-min purge with nitrogen to eliminate any oxygen and prevent the inhibition of free radicals. Subsequently, the initiator solution was added to the monomer solution and stirred for 20 s. The resulting mixture was transferred to three plastic tubes using a pipette and immersed in an aqueous solution at 25 °C for 24 h. Afterward, the formed gels were removed from the tubes, cut into uniform cylindrical shapes, and washed with methanol for 24 h using a Soxhlet extractor (Asahi Glass plant Inc., Arao City, Japan) to eliminate any unreacted components. The gels were then air-dried at room temperature and further dried in an oven at 50 °C for 24 h. Finally, the dried gels were ground into a powder using a grinder.

### 4.3. Synthesis of the DMAA Hydrogel

In the hydrogel synthesis process, 2.478 g of DMAA monomers was precisely measured and combined with 0.193 g of MBAA crosslinker and 0.058 g of TEMED accelerator in a 20 mL volumetric flask (refer to Table 3). Distilled water was added to the flask, and the resulting mixture was stirred using a magnetic stirrer. Concurrently, a solution containing 0.057 g of APS initiator was prepared in a separate 5 mL volumetric flask using distilled water. To eliminate any oxygen and prevent the inhibition of free radicals, both the monomer solution and initiator solution underwent a 45-min nitrogen purge. Subsequently, the initiator solution was added to the monomer solution and stirred for 20 s. The resulting mixture was transferred to three plastic tubes using a pipette and immersed in an aqueous solution at 25 °C for 24 h. After the immersion period, the formed gels were extracted from the tubes, shaped into uniform cylindrical forms, and subjected to a 24-h methanol wash using a Soxhlet extractor (Asahi Glass plant Inc., Arao City, Japan) to remove any unreacted components. Following this process, the gels were air-dried at room temperature and further dried in an oven at 50 °C for 24 h. Lastly, the dried gels were finely ground into a powder using a grinder.

### 4.4. Experimental Design

A total of 800 g of moist soil was precisely measured and transferred to a Neubauer pot with a precision of 1/10,000a. Subsequently, varying concentrations (0%, 2%, and 4% *w*/*w*) of DMAPAA/DMAPAAQ and DMAA hydrogels were introduced into the soil. Additionally, CdCl_2_ solutions at concentrations of 0, 2, and 500 mg/kg were incorporated into the soil that only contained the DMAPAA/DMAPAAQ hydrogel. Another experimental group, the DMAPAA/DMAPAAQ and DMAA group, did not include cadmium. Thorough mixing was conducted to ensure the even distribution of cadmium and the hydrogel within the soil matrix. The soil was then allowed to reach a semi-moist state over a span of several days, following which it was divided into two equal portions. To facilitate plant growth, small holes were made in each of the four directions of the pot. Subsequently, four seeds of Swiss chard (*Beta vulgaris* L. var. cicla) were carefully placed in each hole.

### 4.5. Dry Weight of the Vegetables

Following a two-month cultivation period in a greenhouse, the roots of eight Swiss chard plants were harvested by cutting them with scissors. Any soil adhering to the roots was washed away with water. Subsequently, the harvested plants were carefully arranged in envelope bags and subjected to drying in an oven set at 70 °C for several days until complete dryness was achieved. The electronic balance was then employed to measure the dry weights of the Swiss chard.

### 4.6. Plant Digestion

Four plants of similar size were selected, and their dried samples were ground using a magnetic grinder (BMS-A20TP, Biomedical Science Corp., Tokyo, Japan) at 700 revolutions per minute for a duration of 15 min. Subsequently, approximately 50 mg of each crushed sample was carefully weighed and recorded. Following this, 2 mL of nitric acid and 0.5 mL of hydrogen peroxide were added to each centrifuge tube, and the mixture underwent digestion using a heat block incubator (DTU-2BN, Taitec Corp., Saitama, Japan). The digestion process was initiated at 80 °C for 30 min, followed by a gradual increase in temperature to 120 °C for 2 h, and was then left overnight in a fume hood. After digestion, the resulting solution was filtered through filter paper and adjusted to a volume of 25 mL in a volumetric flask. Finally, the concentration of various elements in the vegetables was determined using ICP.

### 4.7. Experiment on Cadmium Precipitation from the Soil

First, we prepared two separate 40 milliliter solutions by utilizing 1 mol/L hydrochloric acid (HCl) and adjusted their pH values to 2 and 7. Afterward, we incorporated 4 g of soil into each solution, achieving a solid-to-liquid ratio of 1:10. We stirred the mixture on a shaker at 25 °C for 18 h to ensure comprehensive and uniform mixing. Following this, we filtered the mixture through a 0.22 micrometer syringe filter to obtain a clear solution. Lastly, we utilized inductively coupled plasma (ICP) mass spectrometry to measure the concentration of cadmium in the solution.

### 4.8. Characterization

The concentration of the heavy metal cadmium in the solution was determined through ICP (SPS-3500, Shimadzu Corp., Kyoto, Japan). The daily growth of the vegetables was monitored and recorded using a high-resolution camera (Xiaomi12 Technology Co., Ltd., Beijing, China). Additionally, the elemental concentration of the vegetables was analyzed using ICP. The pH of the soil was measured with precision using a pH meter (Hanna Groline Soil pH Tester HI981030, Hanna Instruments, Woonsocket, RI, USA). Further characterization of the soil properties was conducted through EDX analysis (EDX-7000, Shimadzu Corp., Kyoto, Japan).

## Figures and Tables

**Table 1:** Comparison of the adsorption of the heavy metal cadmium by different materials.

Adsorbent	Heavy Metal	Adsorption Capacity (mg/g)	Reference
Amino-functionalized silica	Cd	18.25	Heidari et al. [40]
MnO_2_-loaded resin	Cd	21.5	Dong et al. [41]
Corncob-supported Al-Mn oxides	Cd	45.6	Zheng et al. [42]
Water hyacinth BC	Cd	70.3	Zhang et al. [43]
Na-Bentonite	Cd	30	Ayuso et al. [44]
Nano-hydroxyapatite/ chitosan composite	Cd	92	Salah et al. [45]
Rice husk ash	Cd	3.04	Srivastava et al. [46]
DMAPAA/DMAPAAQ gel	Cd	121	This work

**Table 2:** Synthesis conditions for the DMAPAA/DMAPAAQ hydrogel.

Materials	Component Type	Molar Weight (g/mol)	Concentration (mol/m^3^)	Mass (g)
DMAPAA	monomer	156.22	500	1.953
DMAPAA-Q	monomer	206.71	500	2.584
MBAA	linker	154.17	50	0.193
TEMED	accelerator	116.21	20	0.058
APS	Initiator	228.19	20	0.114

**Table 3:** Synthesis conditions for the DMAA hydrogel.

Materials	Component Type	Molar Weight (g/mol)	Concentration (mol/m^3^)	Mass (g)
DMAA	monomer	99.13	1000	2.478
MBAA	linker	154.17	50	0.193
TEMED	accelerator	116.21	20	0.058
APS	Initiator	228.19	20	0.114

## Data Availability

All data and materials are available on request from the corresponding author. The data are not publicly available due to ongoing research using a part of the data.

## Supplementary Materials

The following supporting information can be downloaded at: https://www.mdpi.com/article/10.3390/gels10050348/s1, Figure S1: Dry weight of the aboveground parts of vegetables after two different types of hydrogels were added to the soil.
