# Supplementary material for: Functional Hydrogels Promote Vegetable Growth in Cadmium-Contaminated Soil
Source: Gels. 2024 May 20;10(5):348. doi: 10.3390/gels10050348 (PMC11121211; doi:10.3390/gels10050348)
Supplement: Supplementary file 1 [file gels-10-00348-s001.zip › gels-2956229-supplementary.pdf]

Supplementary Materials

Functional Hydrogels Promote Vegetable Growth in Cadmium-Contaminated Soil

Jin Huang [1], Takehiko Gotoh [1,*], Satoshi Nakai [1] and Akihiro Ueda [2,*]

[1] Graduate School of Advanced Science and Engineering, Hiroshima University, 1-4-1 Kagamiyama, Higashi-Hiroshima 739-8527, Hiroshima, Japan; hj520618618@outlook.com (J.H.); sn4247621@hiroshima-u.ac.jp (S.N.)

[2] Graduate School of Integrated Sciences for Life, Hiroshima University, 1-4-1 Kagamiyama, Higashi-Hiroshima 739-8527, Hiroshima, Japan

* Correspondence: tgoto@hiroshima-u.ac.jp (T.G.); akiueda@hiroshima-u.ac.jp (A.U.); Tel.: +81-82-424-7720 (T.G.); +81-82-424-7963 (A.U.)

We utilized an electronic balance to measure the dry weight of the aboveground parts of the vegetables (Figure S1). A progressive increase in dry weight was observed corresponding to the escalating content of DMAPAA/DMAPAAQ gel. Specifically, with the addition of water gel at a concentration of 4%, the maximum dry weight of the vegetables reached 1.46 g, surpassing the value observed in the absence of gel. This unequivocally demonstrates that the incorporation of gel significantly enhances vegetable yield, creating favorable conditions for plant growth and development. In stark contrast, the addition of DMAA gel revealed a distinct decreasing trend in vegetable dry weight as the gel content increased. This trend suggests that DMAA, functioning as a non-ionic gel, does not promote plant growth and appears to exert a negative impact on vegetable dry weight. This sharp disparity underscores the superior efficacy of DMAPAA/DMAPAAQ gel in promoting plant growth and increasing yield. These observational results provide crucial experimental support for a more comprehensive understanding of the diverse effects of different types of gel on plant growth.

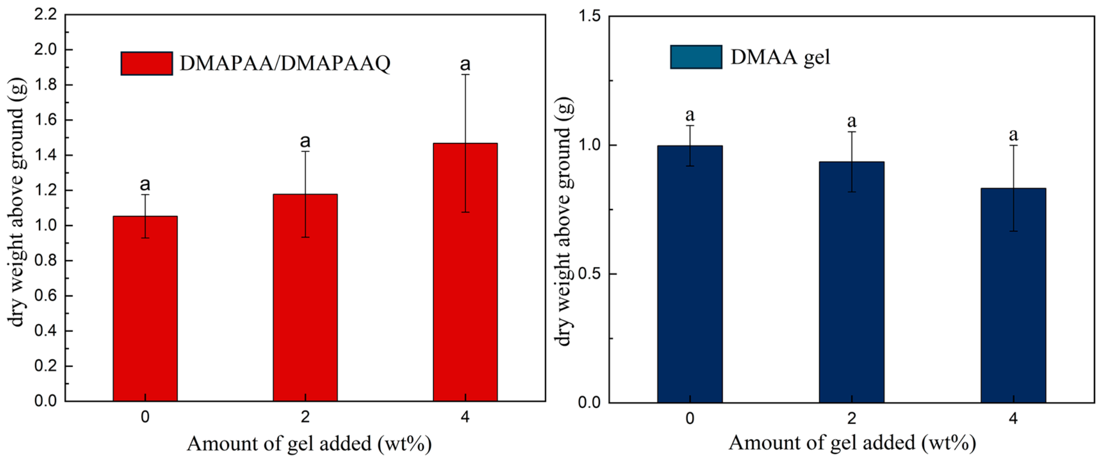

Figure S1. Dry weight of the aboveground parts of vegetables after two different types of hydrogels were added to the soil.
